# Tumor Microenvironment in Cancer Biology: A Comprehensive Review of Stromal, Immune, and Vascular Components Driving Malignancy

**DOI:** 10.7759/cureus.100320

**Published:** 2025-12-29

**Authors:** Sushma T A, Sudarshan Gupta, Priyanka Kiyawat, Yashodha Darshan, Kumar Sambhav, Saiyad Shabbirali Shamshadali

**Affiliations:** 1 Department of Pathology, Shri Atal Bihari Vajpayee Medical College and Research Institute, Rajiv Gandhi University of Health Sciences, Bengaluru, IND; 2 Department of Pathology, Virendra Kumar Sakhlecha Government Medical College, Neemuch, IND; 3 Department of Pathology, Mahatma Gandhi Memorial Medical College, Indore, IND; 4 Department of Pathology, Sri Chamundeshwari Medical College Hospital & Research Institute, Channapatna, IND; 5 Department of Anatomy, All India Institute of Medical Sciences, Bilaspur, Bilaspur, IND; 6 Department of Anatomy, Dr. N.D. Desai Faculty of Medical Science and Research, Dharmsinh Desai University, Nadiad, IND

**Keywords:** angiogenesis, extracellular matrix, immune evasion, stromal cells, tumor microenvironment

## Abstract

The tumor microenvironment (TME), comprising the extracellular matrix (ECM), stromal cells, immune cells, and vascular components, plays a decisive role in cancer growth, metastasis, and treatment response. Although cancer research has traditionally focused on tumor cells, increasing evidence shows that the TME actively influences tumor behavior. This narrative review synthesizes recent findings on the stromal, immune, and vascular elements of the TME, drawing on advanced approaches such as single-cell RNA sequencing and AI-assisted imaging. Cancer-associated fibroblasts (CAFs) promote tumor progression through ECM remodeling and immune suppression, while vascular abnormalities limit drug delivery and contribute to therapeutic resistance. Emerging TME-targeted strategies, including anti-angiogenic agents, immune checkpoint inhibitors, and stromal-directed therapies, show promise but remain challenged by TME heterogeneity and tumor adaptability. The evidence indicates that targeting the TME represents a major shift in cancer therapy and offers important opportunities to develop more effective and personalized treatment strategies.

## Introduction and background

The tumor microenvironment (TME) refers to the complex ecosystem surrounding cancer cells, comprising immune cells, stromal cells, blood vessels, and the extracellular matrix (ECM) [1]. This environment continuously interacts with tumor cells and profoundly influences how cancers grow, spread, and respond to treatment. Understanding the TME is important because tumors cannot behave independently; they rely on signals and support from their surrounding environment to survive and progress. A simple way to think about this is to imagine a plant: just as a plant cannot grow without soil, nutrients, and climate, a tumor cannot develop without the microenvironment that nourishes and protects it.

Unlike tumor cells themselves, which have traditionally been the main focus of cancer research, the TME is coming to be understood as a remarkable factor in the growth, spread, metastasis, and treatment resistance of tumors [2]. The TME impacts nearly all cancer biology, such as the initiation of tumorigenesis, secondary tumors, and treatment resistance [3]. This knowledge has transformed the paradigm of cancer research and therapy strategies, and it has become evident that therapies are needed to target the surrounding TME molecular and cellular components, in addition to the tumor cells themselves. Recent high-impact reviews published between 2023 and 2025 have further expanded understanding of stromal, vascular, and immune components of the TME [4-6]. These articles highlight the dynamic and multifaceted interactions within the TME but often focus on isolated pathways or single cellular compartments. In contrast, the present review integrates stromal, vascular, immune, therapeutic, and mechanistic aspects into a unified synthesis. By bringing together these diverse dimensions, this review provides a broader and more clinically relevant overview of the TME landscape.

Cancer research has been historically focused on cancerous cells at the expense of the TME [4]. However, it has emerged in recent decades that tumor cells do not live in a vacuum, but rather, they are in a dynamic interaction with the surrounding environment. The stromal, immune, and vascular elements of TME contribute to the malignant characteristics of the cancer that not only affect the local expansion of the tumors but also the ability of cancerous cells to proliferate and avoid immune system recognition [5]. Initial investigations carried out in the 1970s showed that the stromal compartment (a pool of fibroblasts and endothelial cells) was critical in tumor development [6]. The development of molecular biology, imaging methods, and computational modeling has led scientists to realize that these interactions are much more complicated than originally thought [7]. A major aspect of cancer’s pathophysiology is characterized by tumor development and resistance, of which TME is a significant component.

**Figure 1:**
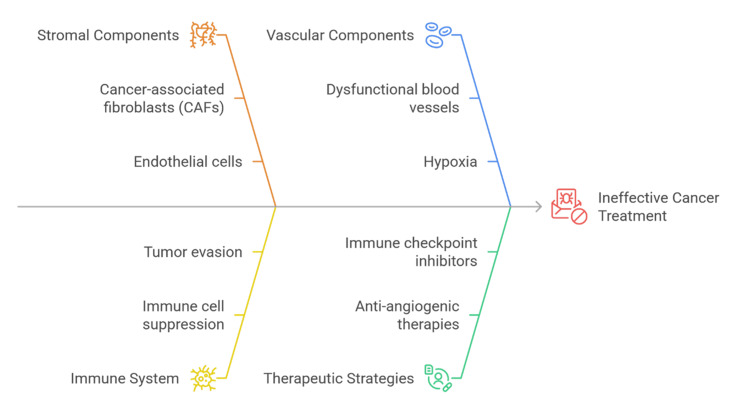
Overview of the elements that make up the TME CAF, cancer-associated fibroblast; TME, tumor microenvironment Created by authors using Napkin AI software

Nonhomogeneous cells that comprise the TME include ECM proteins, endothelial cells, immunological cells, adipocytes, and cancer-associated fibroblasts (CAFs) [8]. They do not merely passively monitor tumor biology. CAFs are essential for tumor development and invasion [9]. They produce the growth factors and cytokines that promote angiogenesis, invasion, and migration of tumor cells, and survival of cancer cells [10]. Additionally, these stromal cells offer an immunosuppressive environment that draws myeloid-derived suppressor cells (MDSCs) and regulatory T cells (Tregs), which weaken the immune response and encourage tumor growth to evade immune surveillance [11]. In addition, the TME’s vasculature is essential for supplying oxygen to malignancies and the nutrients they need. However, the assembly and job of the TME’s vasculature are often disrupted, leading to hypoxic zones that exacerbate tumor aggressiveness and resistance to treatment [12]. Figure 1 shows the main elements of the TME and how they assist in cancer development.

The TME affects not just when cancer first appears but also how effectively a patient reacts to therapy. The TME sends signals to tumor cells that are vital to their survival and growth [13]. The delivery and effectiveness of traditional treatments, including chemotherapy and radiation, are impacted by the TME’s altered vasculature, ECM, and immune cell infiltration. Tumors have an aberrant blood supply, which results in ineffective drug delivery, and immune-suppressive cells interfere with immunotherapies [14]. Furthermore, the TME can respond to the selective pressure of therapy, which helps develop therapeutic resistance. Consequently, TME targeting has emerged as a new treatment option in an attempt to address the shortcomings of conventional cancer therapies [15].

The increased knowledge of the TME has catalyzed the creation of a number of treatment approaches geared at attacking its diverse elements. The ability to normalize the abnormal blood vessels in tumors using anti-angiogenic therapies has demonstrated some success in preclinical models, but clinical success has been minimal [16]. When immune checkpoint inhibitors (ICIs) were developed, immunotherapies revolutionized the therapy for cancer, which targets the tumor’s immune evasion by blocking immune checkpoints, but their success is frequently hampered by the immunosuppressive microenvironment [17].

Therapies that target stroma, which seek to interfere with the supportive activities of the TME [18], are also being studied. Yet, the heterogeneity of the TME is a major obstacle to these treatments, since various tumors have different TME compositions and dynamics. Moreover, side effects on normal tissues can cause unwanted outcomes, which makes the clinical use of TME-targeting therapies more difficult [19]. Despite this, the TME is a promising option for developing new cancer treatments.

Objectives of the review

This narrative review aims to examine how vascular, immunological, and stromal factors contribute to tumor growth and metastasis. It explores the intricate interactions among these components and their collective influence on malignancy. In addition, the review summarizes existing and emerging therapeutic approaches that target the TME, outlining their advantages, limitations, and clinical challenges. By synthesizing current evidence, this narrative review provides a broad and comprehensive understanding of the TME and highlights how focusing on its key components may improve treatment outcomes for cancer patients.

## Review

Components of the TME’s stroma

The stromal compartment of the TME plays a crucial role in cancer development and metastasis [20]. Its major cellular constituents include fibroblasts, myofibroblasts, and ECM components. Under tumor-derived signals, resident fibroblasts differentiate into CAFs [21], which become central regulators of ECM structure and function. Through the secretion of growth factors, cytokines, and enzymes such as matrix metalloproteinases (MMPs), CAFs actively remodel the ECM, promoting tumor growth, motility, and invasion [16,22].

The ECM consists of glycoproteins, including fibronectin, collagen, and elastin, which provide mechanical support to tissues. However, CAF-driven ECM remodeling alters this architecture, creating a more permissive environment for cancer cell movement [18]. MMP-mediated degradation further facilitates tumor migration and is a key driver of metastasis [23]. Changes in ECM stiffness also influence tumor phenotype; increased matrix rigidity activates integrins and mechanotransduction pathways that enhance cancer cell proliferation, survival, and invasiveness [10].

Myofibroblasts represent another important stromal component. Unlike quiescent fibroblasts, myofibroblasts exert contractile forces on the surrounding matrix and secrete pro-tumorigenic factors, thereby supporting ECM remodeling and promoting malignancy [21,24]. Their paracrine interactions with tumor cells contribute to a progressively rigid and supportive TME, reinforcing metastatic potential and highlighting the importance of targeting stromal elements to impede tumor progression.

**Figure 2:**
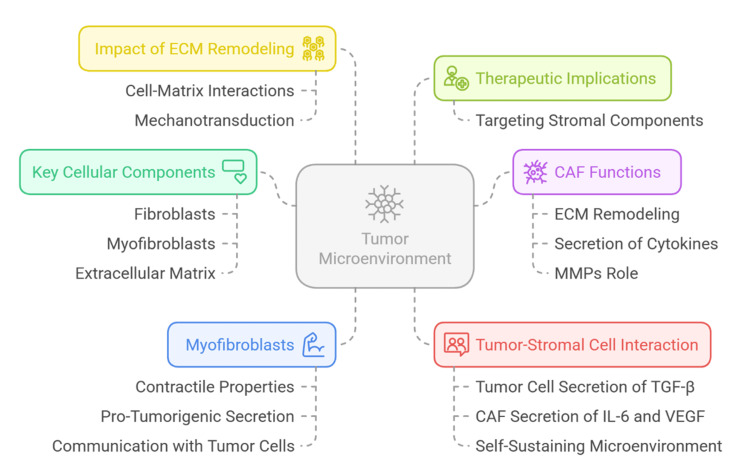
Tumor-stromal interactions and the contribution of CAFs in ECM remodeling and metastasis CAF, cancer-associated fibroblast; ECM, extracellular matrix; MMP, matrix metalloproteinase; TGF-β, transforming growth factor-beta; VEGF, vascular endothelial growth factor Created by authors using Napkin AI software

In addition to shaping a pro-tumorigenic milieu, tumor cells secrete transforming growth factor-beta (TGF-β), which further drives fibroblast-to-CAF differentiation [24]. Dysregulation of the complement cascade within this environment promotes chronic inflammation, tumor cell survival, and immune evasion [25]. Activated CAFs also release angiogenic and inflammatory mediators, including vascular endothelial growth factor (VEGF) and IL-6, establishing a self-sustaining microenvironment that supports tumor survival, metastasis, and therapeutic resistance [26]. This dynamic stromal-tumor interaction forms a permissive niche for cancer development and underscores the therapeutic potential of targeting stromal components [27]. The interconnected roles of CAFs, ECM remodeling, and mechanical signaling are illustrated in Figure 2, emphasizing their contribution to metastasis and matrix reorganization.

Immune cells linked to tumors

Immune cells within the TME possess both antitumor and pro-tumor functions. Key populations include dendritic cells, macrophages, and cytotoxic CD8⁺ T cells, which play central roles in tumor surveillance and tumor cell elimination [28]. Although CD8⁺ T cells are essential for antitumor immunity, their activity is frequently diminished as the TME fosters immunological tolerance [15]. Tumors achieve immune escape partly by recruiting immunosuppressive cell populations, most notably MDSCs and Tregs, which inhibit T cell activation and weaken dendritic cell function [5]. Through these mechanisms, MDSCs and Tregs create an immunosuppressive environment that permits tumor progression [29].

Tumor-associated macrophages (TAMs) represent another major immunosuppressive component of the TME. Shifting toward an M2-like phenotype, TAMs secrete cytokines such as IL-10 and TGF-β that promote tumor growth, metastasis, and immune escape [30]. In contrast, tumor-infiltrating lymphocytes, particularly CD8⁺ T cells, are generally considered positive prognostic indicators because their presence reflects ongoing immune engagement. However, their function can be impaired by immune checkpoint molecules such as CTLA-4 and PD-1, which dampen effector T cell activity. Many tumors overexpress PD-L1 and other checkpoint ligands that bind PD-1 on T cells, preventing activation and enabling immune evasion [31].

Despite the essential role immune cells play in tumor control, tumors develop multiple strategies to suppress or bypass these defenses, underscoring the centrality of immune evasion in cancer progression. ICIs aim to reverse this suppression by disrupting PD-1/PD-L1 interactions and restoring T cell function, yet resistance within the TME remains a major therapeutic challenge. Table 1 summarizes the diverse and often opposing roles of immune cells in the TME, illustrating how immune manipulation enables tumor survival and why these cells remain critical targets for therapeutic intervention.

**Table 1:** Role of immune cells in tumor progression, immune evasion mechanisms, and therapeutic implications MDSC, myeloid-derived suppressor cell; TAM, tumor-associated macrophage; TGF-β, transforming growth factor-beta; TIL, tumor-infiltrating lymphocyte; TME, tumor microenvironment; Treg, regulatory T cell

Immune cell type	Function in tumor development	Mechanisms of immune evasion	Therapeutic implications	References
Tregs	Promotes immunological tolerance by inhibiting effector T cell activity	Tumor cell recruitment and effector T cell suppression	Depletion of Tregs or inhibition of Treg function to boost antitumor immune responses	[6]
TAMs	M2 phenotype-polarized, encouraging angiogenesis, metastasis, and tumor growth	Immune system is suppressed when TGF-β and IL-10 are secreted	Targeting TAM polarization and cytokine secretion to enhance antitumor immunity	[7]
TILs	A favorable prognostic indicator, an immune response against the tumor	TME exhaustion brought on by inhibitory molecules such as CTLA-4 and PD-1	Immune checkpoint medications like those that block PD-1/PD-L1 can prevent TIL exhaustion by reviving the immunological response	[10]
Dendritic cells	Antigen presentation and activation of T cells	TME-driven suppression reduces dendritic cell function and antigen presentation	Strategies to improve dendritic cell activity and enhance T cell activation	[12]
CD8⁺ T cells (cytotoxic)	Crucial for tumor surveillance and elimination of tumor cells	TME induces immune tolerance, limiting activation of T cells and cytotoxic function	T cell function can be restored by using immunological checkpoint inhibitors, like anti-PD-1 and anti-CTLA-4	[26]
MDSCs	Inhibit dendritic and T cells, which aids in tumor development and immune evasion	MDSC recruitment to TME via tumor-secreted signals (e.g., cytokines), suppressing antitumor immunity	Targeting MDSCs to reduce their immunosuppressive role and enhance T cell activity	[26]

Angiogenesis and vascular components

Angiogenesis, the formation of new blood vessels from existing ones, is a major driver of tumor growth and metastasis. Within the TME, stromal and tumor cells release pro-angiogenic factors such as fibroblast growth factor (FGF), VEGF, and angiopoietins, creating conditions that promote endothelial cell migration and proliferation [32]. These signals stimulate the formation of new vessels that support tumor expansion; however, the vasculature formed in tumors is typically disorganized, structurally abnormal, and functionally inefficient [33]. This aberrant vascular network is a defining feature of the TME and is closely linked to cancer progression and dissemination.

Endothelial cells lining tumor-associated blood vessels respond directly to pro-angiogenic molecules produced by surrounding stromal and cancer cells, driving vessel sprouting and remodeling [34]. Although angiogenesis provides tumors with essential oxygen and nutrients, the resulting vasculature is highly irregular and leaky, impairing the effective delivery of chemotherapeutic agents and contributing to poor therapeutic outcomes [35]. This “vascular barrier” is a major obstacle in oncology and plays a key role in resistance to chemotherapy and other systemic treatments [17]. Pericytes, which normally stabilize vessels, are often lost or detached in the TME, further promoting vascular fragility and leakiness; this destabilization is associated with increased tumorigenesis and metastatic potential [11].

Hypoxic regions arise as a consequence of abnormal vasculature and further intensify tumor aggressiveness. Hypoxia activates hypoxia-inducible factors (HIFs), which upregulate genes involved in angiogenesis, metabolic adaptation, and cell survival [6,34]. The combination of hypoxia and vascular irregularity therefore reinforces tumor progression while simultaneously limiting drug penetration.

The influence of angiogenesis on therapy is multifaceted: although it sustains tumor growth by supplying blood, its dysfunctional nature severely compromises drug delivery [19]. Poor perfusion and vascular leakiness hinder the distribution of chemotherapeutic agents, promoting resistance and treatment failure [35]. Anti-angiogenic therapies aimed at inhibiting or normalizing tumor blood vessels have shown promise in preclinical settings, but clinical outcomes remain modest due to incomplete vascular normalization and limited impact on tumor burden [23]. Nevertheless, angiogenesis remains a critical determinant of tumor growth, metastasis, and therapy resistance. Strategies targeting vascular remodeling, enhancing drug delivery, or inhibiting angiogenic pathways continue to represent important avenues for developing more effective cancer treatments [15,36].

Hypoxia and its impact on tumor progression

Hypoxia is a defining feature of the TME and plays a critical role in tumor progression and treatment resistance [4]. As tumors expand beyond the capacity of their blood supply, regions of low oxygen tension develop [37], triggering adaptive responses that enhance tumor survival and aggressiveness. Central to these responses are HIFs, particularly HIF-1α and HIF-2α, which regulate how tumor cells respond to oxygen deprivation [22]. Under hypoxic conditions, HIFs stabilize, translocate to the nucleus, and activate genes involved in angiogenesis, glycolysis, and cell survival. Among the most significant targets of HIF activation is VEGF, which drives angiogenesis to improve nutrient and oxygen delivery [31].

Hypoxia also contributes to extensive metabolic reprogramming. HIF-1α upregulates glycolytic enzymes such as glucose transporters and hexokinase 2, promoting a shift toward anaerobic glycolysis, a hallmark of tumor metabolism known as the Warburg effect [38]. This metabolic flexibility enables tumor cells to survive and proliferate even in oxygen-poor conditions, thereby promoting aggressiveness and invasion [12]. In addition, hypoxia induces MMPs, facilitating ECM degradation and enhancing tumor cell invasion and metastasis [15].

**Figure 3:**
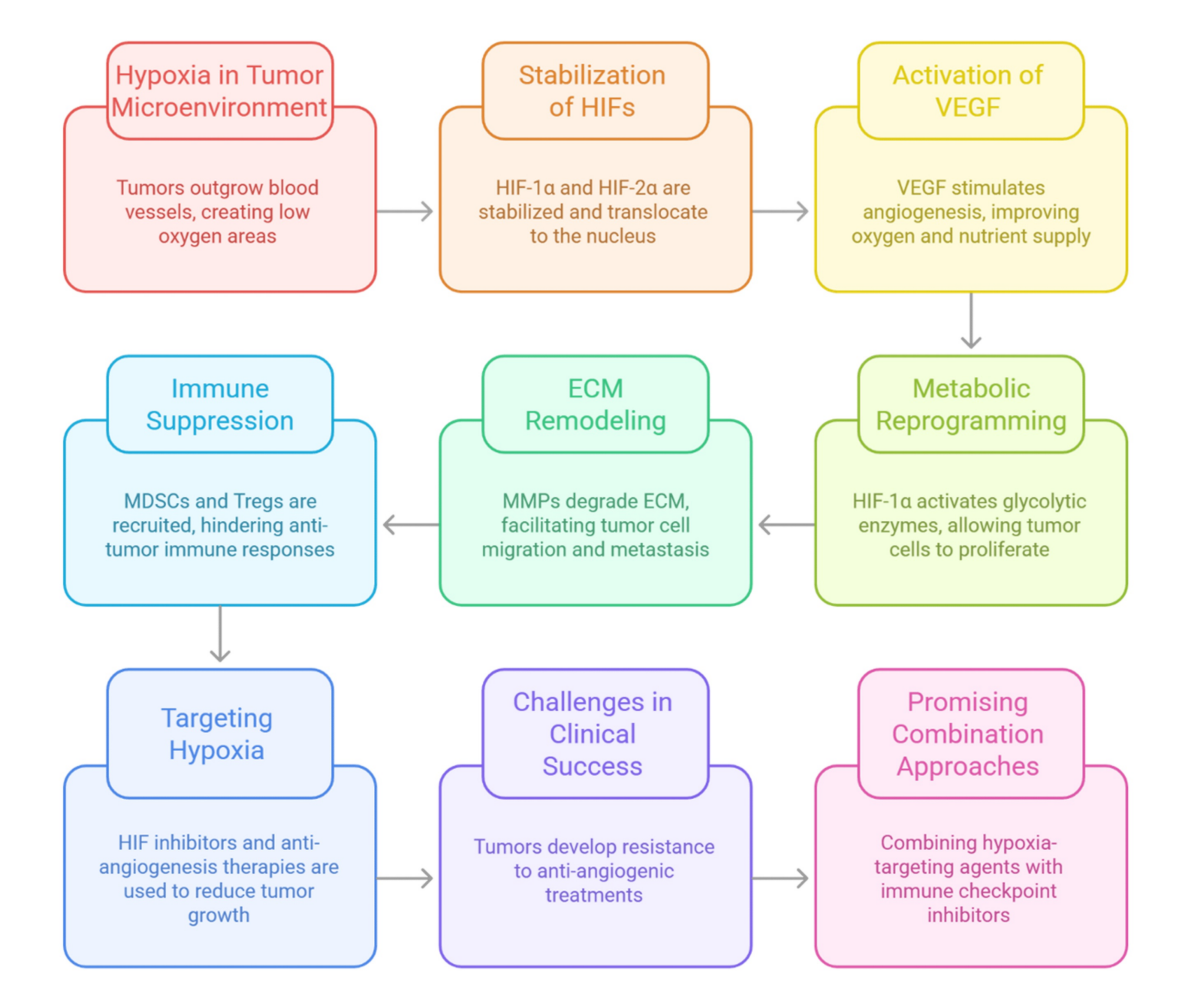
Role of hypoxia in tumor progression and therapy resistance ECM, extracellular matrix; HIF, hypoxia-inducible factor; MDSC, myeloid-derived suppressor cell; MMP, matrix metalloproteinase; Treg, regulatory T cell; VEGF, vascular endothelial growth factor Created by authors using Napkin AI software

The immunological consequences of hypoxia further reinforce tumor progression [39]. Hypoxic conditions stimulate the recruitment and activation of immunosuppressive populations, including MDSCs and Tregs, which dampen antitumor immunity [26]. Tumor cells in hypoxic niches also upregulate PD-L1, allowing them to escape immune surveillance by promoting T cell exhaustion and immune evasion [40]. These combined effects highlight how hypoxia orchestrates metabolic, structural, and immunological changes that collectively promote tumor progression and therapy resistance. Figure 3 illustrates the multifaceted impact of hypoxia on tumor biology and treatment outcomes.

Targeting hypoxia is an exciting approach to cancer therapy. Various HIF inhibitors are in development, many of which have demonstrated preclinical activity in inhibiting HIF-1α and limiting tumor growth by suppressing angiogenesis and metabolic adaptation [40]. Bevacizumab, an anti-angiogenic medication that inhibits VEGF, is another tactic used to improve drug delivery and restore normalcy to the tumor’s vascular network. However, the dynamic and complex structure of the TME has hampered the effectiveness of anti-angiogenesis medicines in real life, and many cancer cells develop resistance to these therapies [27]. To restore tumor vasculature, improve immune cell infiltration, and reactivate antitumor immunity, one effective method for overcoming resistance has been verified to be the combination of inhibitors of immune checkpoints and hypoxia-targeting medications [35].

Tumor-stromal interactions and ECM remodeling

The dynamic ECM within the TME plays a critical role in regulating tumor growth and metastasis. Beyond providing mechanical support, the ECM influences essential cellular processes such as invasion, migration, and survival [15]. CAFs are the primary drivers of ECM remodeling; they secrete ECM proteins and alter matrix stiffness, creating conditions that facilitate tumor progression [8]. Increased ECM rigidity activates integrins and downstream signaling pathways that enhance cancer cell motility, invasion, and metastatic potential [23]. MMPs (MMP-2, MMP-9, and MMP-14) further degrade key ECM components, including collagen, fibronectin, and laminin, thereby promoting tumor invasion and metastasis [4]. ECM degradation also releases bioactive fragments capable of stimulating growth factors such as FGF and VEGF, contributing to angiogenesis [17]. Although ECM remodeling allows immune cells to infiltrate the tumor, it simultaneously generates an immunosuppressive environment that favors tumor progression [33].

CAFs play a central role in sustaining ECM remodeling. In response to tumor-derived signals, CAFs continue producing ECM components and reinforcing a feedback loop that accelerates tumor expansion [9]. They also secrete pro-tumorigenic cytokines such as TGF-β and IL-6, which promote angiogenesis and immune suppression [11]. Additionally, CAFs shape the immune microenvironment by polarizing macrophages and recruiting MDSCs, further facilitating immune evasion [12]. Therapeutic strategies targeting the ECM and stromal components are currently under investigation. Although MMP inhibitors aim to block ECM degradation and limit tumor migration, their clinical benefits have been limited [30]. Targeting TGF-β signaling within CAFs is a promising approach, as it may reduce ECM remodeling and enhance antitumor immunity [3]. Combining CAF-directed therapies with ICIs is also under exploration, with the goal of improving immune cell infiltration and strengthening treatment responses [18].

Immune system contributions to tumor progression

The immune system is essential for both promoting tumor growth and protecting the host against tumor cells. Dendritic cells, cytotoxic T lymphocytes (CTLs), and natural killer (NK) cells work together to eradicate cancer cells under normal conditions. However, malignancies typically use immune evasion strategies to grow and spread without triggering the immune system. TAMs, which are initially attracted to the tumor location, undergo reprogramming to adopt the M2 phenotype [41]. TGF-β and IL-10 are examples of immunosuppressive cytokines released by M2 TAMs that promote tumor development, angiogenesis, and metastasis [27]. Through immune evasion tactics, this M2 polarization promotes tumor growth and improves immune suppression by preventing cytotoxic T cell activity [20].

PD-1 and CTLA-4 antibodies (ICIs) are among the most recent developments in cancer immunotherapy [6]. T cells must fulfill their obligations to eliminate the tumor. ICIs stop the immunological checkpoints from communicating with one another. An important concern is the immunosuppressive TME, even though it has been demonstrated that immune checkpoint blockade resistance presents a major obstacle to the treatment of several cancers, including non-small cell lung cancer and melanoma [42]. It is supported by the existence of Tregs and MDSCs, which inhibit the T cell response, making immunological tolerance even harder to maintain and decreasing the effectiveness of immunotherapy [26]. Furthermore, in order to overcome immune suppression within TME, novel technologies such as ICIs, CAR-T cell therapy, and anti-angiogenic medicines are being developed [43]. These technologies have shown early promising activity in hematological malignancies, with solid tumors being studied in clinical trials [13]. Table 2 illustrates the immune cells in immune suppression under TME and cancer formation.

**Table 2:** Immune cells in the TME and their roles in tumor progression CAR, chimeric antigen receptor; CTL, cytotoxic T lymphocyte; MDSC, myeloid-derived suppressor cell; NK, natural killer; TAM, tumor-associated macrophage; TGF-β, transforming growth factor-beta; TME, tumor microenvironment; Treg, regulatory T cell

Immune cell type	Role in tumor progression	Associated mechanism	Key references
Tregs	Reduce the immune response to malignancies and inhibit T cell effectors	Cytotoxic T cell suppression and immunological tolerance	[4]
CAR T cells	Focusing on tumor antigens by reprogramming, showing promise in hematological cancers	Tumor antigen targeting and immune response activation	[10]
NK cells	Find and eliminate tumor cells through innate immune mechanisms	Tumor cell recognition and cytotoxicity	[11]
MDSCs	Dampen the immune system by inhibiting dendritic and T lymphocytes	Promote tumor immune tolerance	[12]
Dendritic cells	T cells become active by processing and presenting antigens	T cell activation and antigen presentation	[23]
CTLs	Eliminate tumor cells through direct cytotoxicity	Tumor surveillance and immune response	[30]
TAMs	Release immunosuppressive cytokines to encourage tumor development, angiogenesis, and metastasis	M2 polarization and the release of IL-10 and TGF-β	[41]

CAFs

A CAF is necessary for the TME and is also important for immune regulation, tumor development, and metastasis [42]. Tumor-derived signals like TGF-β and PDGF activate these fibroblasts together with endothelial cells and pericytes. CAFs undergo phenotypic changes upon activation, which allows them to contribute to immunological suppression, metabolic reprogramming, and ECM remodeling [43]. One of CAFs’ main functions in TME is ECM remodeling, where they make the surrounding environment more mechanically rigid and provide a scaffold to aid in the cancer cells' invasion and migration [44]. Furthermore, CAFs release growth factors required for tumor growth, such as VEGF, FGF, and IL-6, which support angiogenesis, tumor cell survival, and proliferation [16]. These secreted factors not only aid in the structural soundness of the tumor but also aid in immune evasion by recruiting Tregs and MDSCs, which suppress antitumor immune responses [7,26].

Additionally, CAFs undergo metabolic reprogramming, especially via the glycolytic pathway, to generate lactate, which is used as an energy source by tumor cells. Such metabolic symbiosis facilitates additional tumor development and makes therapeutic approaches more complicated [45]. CAFs also play a role in the Warburg effect to promote aerobic glycolysis in stromal and cancer cells, which enables the survival of cancer cells even under hypoxic environments. CAFs are regarded as potential therapeutic targets due to their major role in the enhancement of tumor growth, immune evasion, and metabolic reprogramming [36]. Preclinical models have shown that blocking TGF-β signaling or fibroblast activation protein (FAP) could be used to interfere with CAF activities and limit tumor progression [21]. But the heterogeneity of CAF is an issue, and CAF subtypes can have conflicting effects, so selective targeting is needed. Table 3 demonstrates that CAFs are significant in ECM remodeling, survival of the tumor cells, immune suppression, and metabolic reprogramming, which enhance tumor development and immune escape.

**Table 3:** Role of CAFs in tumor progression and immune modulation CAF, cancer-associated fibroblast; ECM, extracellular matrix; FAP, fibroblast activation protein; FGF, fibroblast growth factor; MDSC, myeloid-derived suppressor cell; TME, tumor microenvironment; Treg, regulatory T cell; VEGF, vascular endothelial growth factor

CAF function	Mechanism	Key factors involved	References
Metabolic reprogramming	Facilitates glycolysis and produces lactate, which tumor cells use for energy, contributing to the Warburg effect	Lactate, glycolysis, and the Warburg effect	[4]
ECM remodeling	Creates a framework for cancer cell invasion and migration by increasing the TME’s mechanical stiffness	ECM proteins and fibroblast activation	[6]
Support tumor cell survival	Releases cytokines, chemokines, and growth factors that encourage tumor development and angiogenesis	IL-6, VEGF, and FGF	[17]
Therapeutic targeting	Targeting TGF-β signaling or FAP to disrupt CAF function and reduce tumor progression	TGF-β, FAP, and CAF depletion	[20]
Immune suppression	Promotes the recruitment of immune system-inhibiting cells and secretes cytokines that inhibit immune responses	Tregs, MDSCs, and TGF-β	[28]

Inflammatory cytokines and chemokines in the TME

The development of tumors and the immune response are influenced by cytokines, chemokines, and essential components of the TME [46]. A proinflammatory milieu that encourages tumor development, metastasis, and immune evasion is probably fostered by such chemicals. Tumors often overexpress chemokines like CXCL12 and CCL2, in addition to TNF-alpha, IL-1, and IL-6 (inflammatory cytokines). Furthermore, tumors aid in the creation of an immune-suppressive milieu that promotes the survival and spread of cancers [47]. Tumor cell survival and proliferation are significantly influenced by IL-6. Cell cycle and anti-apoptotic gene expression are induced by the transcription factor STAT3, which is activated by IL-6 [48]. Furthermore, IL-6 is linked to immunological suppression by promoting the growth of Tregs, which prevent tumor immunosurveillance and effector T cell activity [19]. In a similar vein, TNF-alpha, a cytokine that was once thought to have antitumor properties, has two sides. Tumor cell migration, angiogenesis, and immune system avoidance are all facilitated by the TME's ongoing production of TNF-alpha [21]. Over time, TNF-α may result in immunosuppression. This allows cancerous cells to avoid the immune system’s detection and destruction [49].

Chemokines are also necessary for immune cell penetration into the TME. For instance, CXCL12 draws MDSCs and Tregs and aids in creating a suppressive immunological environment. CCL2, likewise, enhances the attraction of monocytes that differentiate into TAMs [50]. The cytokines produced by these TAMs (especially those in the M2 polarization state) include TGF-beta and IL-10, which impede effector immune cell activation and promote tumor growth and metastasis [26]. This immune infiltration through inflammatory chemokines shows the significance of inflammatory signaling in promoting tumor progression [24]. Since inflammatory cytokines and chemokines are central to tumor progression and immune suppression, they are an attractive therapeutic approach. Blockers of IL-6, like tocilizumab and TNF-α antagonists, are promising in preclinical data and low-stage clinical trials [18]. Also, to increase the immune cells' invasion and augment the performance of cancer treatments, chemokine receptor antagonists that inhibit the recruitment of immunosuppressive cells are under investigation [5].

Metabolic reprogramming in the TME

One characteristic of cancer is metabolic reprogramming, which allows tumor cells to survive the nutrient-deficient hypoxic environment that commonly defines the TME. The Warburg effect (preferential use of glycolysis even when oxygen is available) is one of the most notable changes in metabolism that is observed in tumors. This switch, called aerobic glycolysis, allows tumor cells to produce energy quickly and to supply the biosynthetic intermediates they need to proliferate so fast [27]. Hexokinase 2, the first enzyme in glycolysis, is the most crucial enzyme in the process of converting glucose to glucose-6-phosphate [32]. Glycolysis is not the only metabolic change. A mismatch between reactive oxygen species and antioxidant defenses also contributes to increased oxidative stress in tumor cells [34]. This oxidative stress leads to DNA damage, genetic instability, and tumor progression. Interestingly, metabolic changes also occur in stromal cells, especially CAFs, following tumor-derived signals [24]. CAFs switch to lactate production and secrete the same into the TME. The lactate is taken up by the cancer cells, which can encourage their growth by using it as an additional energy source and metabolic interdependence of stromal and malignant cells [31].

Lactate accumulation produces an acidic environment that reduces immune cells' ability to combat cancer and stops T cells and NK cells from doing their duties [23]. This metabolic synergy between CAFs and tumor cells contributes to immunological suppression in addition to promoting tumor growth, further complicating treatment approaches [33]. As metabolic reprogramming is central to cancer, it has made metabolic pathways in the TME an enticing avenue for cancer treatment. Inhibition of glycolysis with compounds like 2-deoxyglucose has also shown positive results in preclinical trials, in that tumor cells are killed but not normal tissue [28]. Also, there is an investigation of lactate transport-directed therapies or mitochondrial-directed therapies to interfere with the metabolic coupling between tumor and CAFs, providing potential therapeutic options to interfere with therapy resistance and enhance cancer treatment [35]. Table 4 demonstrates the role that metabolic reprogramming of the TME, which includes lactate generation, glycolysis, and oxidative stress, plays in immune evasion, tumor development, and treatment resistance.

**Table 4:** Effect of metabolic reprogramming in the TME on the development of tumors CAF, cancer-associated fibroblast; ICI, immune checkpoint inhibitor; NK, natural killer; ROS, reactive oxygen species; TME, tumor microenvironment

Key component	Description	Impact on tumor progression	Therapeutic strategy	Reference
Oxidative stress	Imbalance between antioxidant defenses and ROS	Contributes to DNA damage, genetic instability, and tumor progression	Targeting ROS production or enhancing antioxidant defenses	[8]
Warburg effect	Even when oxygen is present, cancer cells prefer to produce energy through glycolysis	Enables rapid energy generation and biosynthesis for tumor proliferation	Inhibiting glycolysis (e.g., 2-deoxyglucose) to target tumor cells	[17]
Metabolic symbiosis	CAFs and tumor cells engage in a metabolic symbiosis	Enhances tumor growth and contributes to therapy resistance	Disrupting metabolic coupling between tumor cells and CAFs	[19]
Immune suppression (via lactate)	The acidic environment due to lactate suppresses immune cell activity	Decreased effectiveness of immune cells in attacking tumor cells	Immunocheckpoint inhibitors, inhibitors of PD-1 and PD-L1, are used to stop immunological evasion	[20]
Acidic environment (lactate accumulation)	Lactate accumulation lowers pH in the TME	Impairs immune cell function (e.g., T cells, NK cells), hindering immune response	Combining metabolic targeting with ICIs	[28]
Lactate production by CAFs	CAFs shift toward lactate production, which they secrete into the TME	Tumor cells use lactate as an alternative energy source, fueling growth	Targeting lactate transport to disrupt metabolic coupling	[36]

Emerging therapeutic strategies targeting the TME

Since TME is known to be a key driver of cancer, metastasis, and resistance, it has become a potential therapeutic target. Numerous strategies, including angiogenesis, immunological checkpoint blockage, stromal inhibition, and regulation of inflammatory cytokines, are being considered to tackle the various TME characteristics [51]. Anti-angiogenic medications function by blocking the VEGF pathway, which stimulates the development of fresh blood vessels, to control cancer vasculature and enhance drug delivery [52].

Though tumor adaptability and the introduction of alternative pro-angiogenic pathways have limited their clinical impact, these therapies have demonstrated promise [53]. An example of an ICI that has transformed cancer treatment is antibodies against CTLA-4 and anti-PD-1, which promote immunological responses and enable T cells to eradicate cancerous cells [43]. Even though these medicines have shown promise in the treatment of non-small cell lung cancer and melanoma, among other cancer types, resistance is a significant problem [24].

To circumvent the immune checkpoint blockade resistance, combination treatments are being developed [54]. Anti-angiogenic treatments combined with ICIs, CAF depletion, or inhibition of inflammatory cytokines have high potential in breaking immune suppression and promoting treatment efficacy [4,10]. Also, the metabolic reprogramming of the TME, including glycolysis inhibitors and lactate shuttle inhibitors, is a new strategy to break the metabolic support system that maintains tumor growth [3,55]. Stomal-directed treatment, such as CAFs and ECM, is also explored [56]. The most promising ones are TGF-beta inhibitors and FAP inhibitors in the sense that they can destabilize CAF activity and ECM remodeling [11]. These treatments aim to reduce immunological suppression, chemoresistance, and tumor development [17]. TME heterogeneity is still an issue, though, as CAF subtypes vary in terms of tumor kinds and stages; therefore, very specialized targeting techniques are required to avoid off-target effects.

Limitations and future recommendations

TME research is scarce, which has a detrimental impact on cancer research and treatment development. The first is the dearth of reliable preclinical models that faithfully capture the complexity and heterogeneity of the TME in actual malignancies. The incredibly intricate cellular connections, ECM remodeling, and immune cell infiltrates found in patients cannot be replicated by current models, including 2D cultures and animal models. As a result, research medicines have a limited predictive value, and putting research into clinical practice is difficult. Moreover, due to the significant differences in cellular composition and molecular pathways between TMEs, it is harder to find common treatment targets between different malignancies [57].

Future studies are needed to create a more physiologically relevant preclinical model that recapitulates the full landscape of TME interactions with immune cells, stromal cells, and cancer cells. These models should be patient-specific with an aim to enhance testing of targeted therapies. Establishing individualized treatment plans that take into account each patient's unique characteristics and TME, such as metabolic and immunological profiles, is also necessary. The study of extracellular vesicles (EVs), which are employed for intercellular communication, is one potential research avenue that can be a new source of biomarkers or therapeutic targets to overcome therapy resistance due to TME, which can increase the overall efficacy of treatment [58].

## Conclusions

Developing physiologically relevant preclinical models that accurately capture the full spectrum of TME interactions among stromal, immune, and cancer cells should be a major priority moving forward, as these interconnected components strongly influence tumor survival, invasion, immune evasion, and resistance to conventional therapies. Although emerging TME-targeted approaches, such as angiogenesis inhibitors and immune checkpoint blockers, show promise, their effectiveness is limited by the heterogeneity and adaptive nature of the TME. Combining tumor-specific therapies with strategies that modulate the TME may help overcome these barriers, especially through multi-targeted treatment designs. Future research must therefore focus on improving preclinical models that better reflect TME complexity and patient-specific variability, enabling more precise and durable therapeutic responses. Personalized treatments that incorporate individual metabolic, immune, and stromal characteristics of the TME, along with the exploration of EVs as communication mediators and biomarkers, offer additional avenues for combating TME-driven therapy resistance. Despite this potential, challenges such as persistent immune suppression and tumor plasticity must still be addressed to ensure the successful translation of TME-focused therapies into clinical practice.
